# Fabrication of Ag/ZnO@N-Carbon Core@Shell Photocatalyst for Efficient Photocatalytic Degradation of Rhodamine B

**DOI:** 10.3389/fchem.2022.950007

**Published:** 2022-06-30

**Authors:** Xiaobing Yang, Jiapeng Hu, Junjie Pan, Yongbin Shen, Kejun Cheng

**Affiliations:** ^1^ School of Photovoltaic Materials, Jiangxi New Energy Technology Institute, Xinyu, China; ^2^ Fujian Provincial Key Laboratory of Eco-Industrial Green Technology, Wuyi University, Wuyishan, China; ^3^ Chemical Biology Center, Lishui Institute of Agriculture and Forestry Sciences, Lishui, China

**Keywords:** ZAg/ZnO@N-carbon, rhodamine B, photocatalytic activity, stability, ZIF-8

## Abstract

Photocatalytic degradation method has been recognized as an effective way to eliminate the contamination of environment. However, developing photocatalysts with excellent photocatalytic properties are still a big challenge. In this paper, Ag doped ZnO coating with a layer of N doped porous carbon (Ag/ZnO@N-carbon) was successfully synthesized by using polyvinyl pyrrolidone (PVP) modified ZIF-8 as precursor *via* adsorption, hydrothermal treatment, *in situ* growth and carbonization processes. The physical and chemical properties of all samples were characterized by X-ray powder diffraction (XRD), scanning electron microscope (SEM), electron transmission microscopy (TEM) and so on. The results show that Ag doping does not change the crystallinity of ZnO, but broaden its photo-response property. The coating of N doped carbon can improve the specific surface area of photocatalyst. The photocatalytic activity of all samples was evaluated by degradation of rhodamine B (RhB) solution under UV light irradiation for 25 min. Ag/ZnO@N-carbon exhibits the highest photocatalytic activity for degradation of RhB with a degradation of 98.65%. Furthermore, Ag/ZnO@N-carbon also has high stability. Based on the characterization, possible mechanism for degradation of RhB by Ag/ZnO@N-carbon under UV light irradiation was proposed.

## Introduction

Our economy has dramatically improved due to the rapid development of the industry. However, excessive economic expansion has some negative consequences, such as significant contamination of the environment, which endangers human life (Park et al., 2019; Cheriyan and Choi, 2021; Wang et al., 2021; Chang et al., 2022). In this context, semiconductor photocatalysis has captured intense attention because it can provide environmental-friendly pathways to solve the severe problems of concerned environmental pollutants. Semiconductor photocatalysts, such as LaFeO_3_ (Khan et al., 2020; Khan et al., 2021), ZnIn_2_S_4_ (Swain et al., 2019), g-C_3_N_4_, and ZnO (Qiu et al., 2016; Zhao et al., 2020), have been widely prepared and used to degrade organic containments in drinking water. ZnO has drawn increasing interest among these semiconductor photocatalysts due to its low cost, chemical stability, non-toxicity, and increased photosensitivity (Balachandran and Swaminathan, 2012). However, ZnO’s bandgap and electron-hole recombination rate are relatively wide and high (Sun et al., 2009). It highly limits the practical application of ZnO. Therefore, developing novel photocatalysts with a relatively narrow bandgap and excellent charge carrier separation is essential.

Various techniques, such as doping with other elements, have been used to produce efficient ZnO-based materials for photo-degradation of organic containments (Qi et al., 2017), integrating with narrow bandgap photocatalysts, and designing their morphology (Hong et al., 2009; Uddin et al., 2012). Doing additional elements is an effective technique to extend the bandgap and boost the electron-hole recombination rate of ZnO, resulting in improved photocatalytic activity. Jiang and his colleagues, for example, synthesized a series of Cu-doped ZnO (Cu-ZnO) photocatalysts using a simple hydrothermal technique and looked at the effect of Cu doping on photocatalytic activity Jiang et al. (2019). They discovered that 1.5% Cu-ZnO had the best photocatalytic activity for Rhodamine (RhB) degradation when exposed to UV light. Alam et al. created a variety of Y and V co-doped ZnO (YVZ) nanoparticles using a surfactant-assisted sol-gel method. They tested its photocatalytic activity for the 4-nitrophenol (4-NP), Methylene Blue (MB), and Rhodamine B (RhB) degradation under visible light irradiation Alam et al. (2017). The most active photocatalyst was 3%Y/1%V-ZnO to degrade RhB, MB, and 4-NP. Ahmad and his coworkers successfully synthesized Al-doped ZnO photocatalysts with different Al concentrations through calcination at 500^o^C for 3 h (Ahmad et al., 2013). They discovered that doping ZnO with 4.0 mol%, Al can increase visible light absorption, limit electron-hole pair recombination, and increase ZnO absorptivity. And, the shape of ZnO has a substantial influence on its photocatalytic activity. Yang et al., for example, effectively microwave-heated a dumbbell-shaped ZnO photocatalyst Yang et al. (2010). Their findings demonstrated that Methylene Blue (MB) exhibited higher decolorization and TOC removal efficiency than commercial ZnO powder, at 99.6% and 74.3%, respectively. Tian et al. prepared a novel ZnO photocatalyst by direct calcination of zinc acetate at moderate temperature and used it as a photocatalyst to degrade MO Tian et al. (2012). The results show that ZnO-600 can degrade 98% of MO, superior to P25 TiO_2_ (46%). However, there are very few reports about combining the doping of some other elements method and the designing the morphology of ZnO photocatalyst.

This work synthesized an Ag-doped ZnO (Ag/ZnO) polyhedral structure *via* adsorption and hydrothermal treatment utilizing PVP modified ZIF-8 as a precursor. Then, Ag/ZnO was coated with a layer of N-doped carbon (Ag/ZnO@N-carbon) using *in situ* growth and carbonization techniques. The photocatalytic activity of Ag/ZnO@N-carbon was determined by degrading RhB in the presence of UV light. After five recycling studies, Ag/ZnO@N-carbon showed exceptional photocatalytic activity and high reusability. Finally, a plausible mechanism for the effect of Ag doping on ZnO photocatalytic activity was presented. The synergistic effect of Ag/ZnO and N-doped carbon on RhB degradation has been comprehensively investigated.

## Exoerunebtal Sectuib

### Materials

2-methylimidazole, zinc nitrate hexahydrate [Zn(NO_3_)_2_·6H_2_O], absolute methanol and absolute ethanol were of analytical grade and obtained from Sinopharm Chemical Reagent Co., Ltd. Polyvinyl pyrrolidone (PVP, K30), silver nitrate (AgNO_3_) and rhodamine B (RhB) were analytical grade and purchased from Shanghai Macklin Biochemical Co., Ltd. All reagents were directly used as received without any further treatment. Deionized water was used throughout the experiments. Synthesis of Ag/ZnO@N-carbon core@shell photocatalyst.

In order to make an Ag-doped ZnO coating with a layer of N-enriched carbon (Ag/ZnO@N-carbon), a PVP modified ZIF-8 (PVP/ZIF-8) was used as a precursor. PVP modified ZIF-8 was prepared using the previously modified technique (Liu et al., 2016). First, in 100 ml of 100% methanol, a 10 g of PVP and 4.5 g of 2-methylimidazole were dissolved. Additional solution of methanol (100 ml) containing 3.8 g of Zn(NO_3_)_2_·6H_2_O was promptly added to the solution described above and agitated for 5 min at room temperature to achieve the homogenous straw yellow solution. The combination was then aged for 15 h at 60°C. The synthesized PVP/ZIF-8 was centrifugated, washed ×3 times (100% ethanol), and dried for 10 h at 80°C. Next, to make a homogenous solution, 0.0511 g of AgNO_3_ was dissolved in 5 ml of deionized water and then combined with 25 ml of pure alcohol. The PVP/ZIF-8 (1 g) was mixed in the solution as mentioned earlier and stirred at room temperature for 30 min. To obtain Ag-doped PVP/ZIF-8, the suspension was centrifugated and dried at 80°C for 10 h. To generate Ag-doped ZnO (Ag/ZnO), at 570°C for 40 min the Ag-doped PVP/ZIF-8 was then pyrolyzed at a rate of 2°C/min. Then, at 60°C, 0.5 g of Ag/ZnO was poured into 30 ml of ethanol solution containing 4 g of PVP and 9.1 g of 2-methylimidazole. The product was centrifuged after 3 h, rinsed with ethanol, and dried for 10 h at 80°C. Finally, the above-obtained product was pyrolyzed at 800°C for 30 min with the heating rate of 2°C/min under Ar atmosphere to synthesize the Ag-doped ZnO coating with a layer of N enriched carbon (Ag/ZnO@N-carbon).

### Characterization

To analyze the crystal structures from 20^o^ to 70^o^, a Bruker D8 Advance device was used to obtain XRD patterns through Cu-Kα radiation (λ = 0.15418 nm). SEM pictures were taken with a JSM-7600F apparatus to record the morphology of all as-synthesized samples. TEM and HRTEM pictures were acquired using JEM2100F equipment. On a Micromeritics ASAP 2020 equipment, N_2_ adsorption-desorption isotherms of all as-synthesized materials were examined. Before being examined, materials were degassed for 8 h at 120°C using the BET method and Barrett-Joyner-Halenda (BJH) model, the BET surface area and average pore size were calculated. To calculate the concentration of N-doped carbon on Ag/ZnO@N-carbon, a TG curve was produced on a thermal instrument (Thermo plus EVO2, Japan) with a heating rate of 5°C/min under an air environment. UV-vis absorption spectra were calculated from 200–800 nm using a Shimadzu UV-3100 spectrophotometer. To assess the chemical compositions, XPS analysis was performed on a VG ESCALAB 210 XPS machine equipped with an Mg Ka source.

### Catalytic Activity Testing

By decomposing RhB solution under UV light irradiation, the photocatalytic performance of all generated samples was estimated. A 500-watt mercury lamp served as the UV light source. In one experiment, 50 mg of the photocatalyst was dispersed in 100 ml of a 5 ppm RhB solution. To reduce the influence of photocatalyst adsorption, the suspension solution was magnetically agitated at room temperature for 30 min in the dark before irradiation. At regular irradiation intervals, 5 ml of the suspension was taken from the reaction vessel and centrifuged to remove the photocatalyst. The concentration of RhB solution was determined using a UV-vis spectrophotometer (UV-2600, Shimadzu). The degradation rate of RhB (D_RhB_) was estimated using the following formula:

$$D_{\mathrm{RhB}}=\left(C_{o}-C_{t}\right)\times 100\%÷C_{o}$$

The C_o_ and C_t_ refer to the RhB solution concentration at the start and after exposure to radiation for time “t” in minutes.

## Results and Discussion

### X-Ray Powder Diffraction Patterns Analysis

**FIGURE 1:**
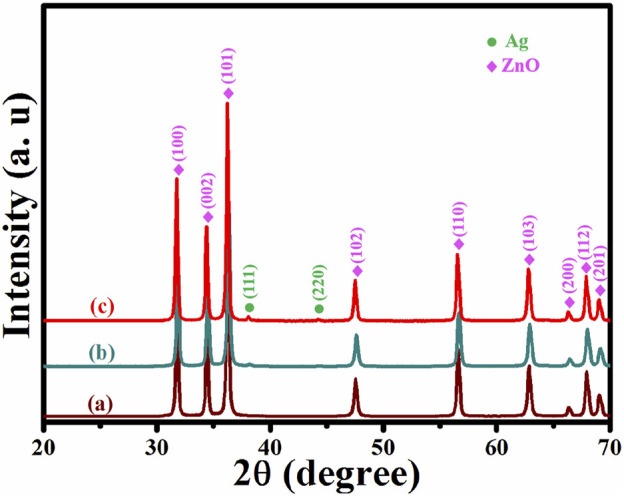
XRD patterns of **(A)** ZnO, **(B)** Ag/ZnO, **(C)** Ag/ZnO@N-cabon.

The XRD patterns of all as-synthesized samples are depicted in Figure 1. The XRD pattern of wurtzite-type ZnO produced from PVP modified ZIF-8 is shown in Figure 1A (JCPDS Card No. 36-1451) (Raoufi and Raoufi, 2009). The XRD patterns of Ag/ZnO are shown in Figure 1B. The Ag/ZnO has two new peaks at 38.11^o^ and 44.30^o^ in comparison to ZnO, which are due to the (111) and (220) reflections of Ag, respectively (JCPDS Card No. 87-0597) (Onkani et al., 2020). Figure 1C depicts the Ag/ZnO@N-carbon’s XRD pattern, which is identical to Ag/ZnO. It demonstrates that coating Ag/ZnO with N-doped carbon does not affect the crystalline structure.

### Scanning Electron Microscope Images Analysis

**FIGURE 2:**
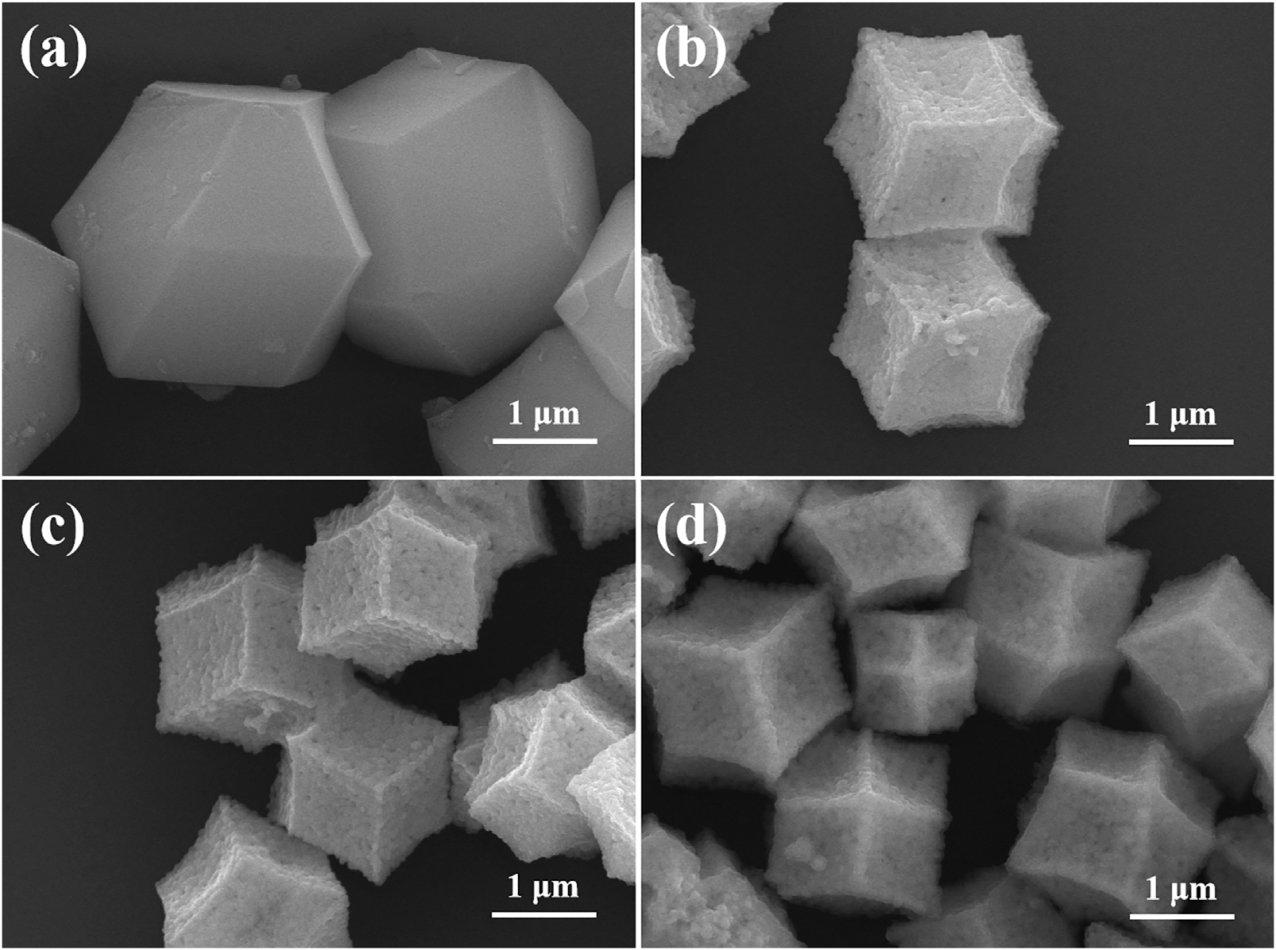
SEM images of **(A)** ZIF-8, **(B)** ZnO, **(C)** Ag/ZnO, **(D)** Ag/ZnO@N-carbon.

The surface morphology was assessed by SEM technology. As shown in Figure 2A, PVP modified ZIF-8 exhibits a polyhedral structure with a particle size of 2.6 μm. Figures 2B,C are the SEM images for ZnO and Ag/ZnO. Both ZnO and Ag/ZnO show the polyhedral structure. However, their particle sizes are smaller than PVP-modified ZIF-8. It is attributed to the pyrolysis of organic ligands of ZIF-8. As shown in Figure 2D the Ag/ZnO@N-carbon exhibits the polyhedral structure. The particle size of Ag/ZnO@N-carbon, on the other hand, is about 2.0 um, which is greater than Ag/ZnO (1.6 um). Ag/ZnO can act as a Zn source to form a layer of ZIF-8 on its surface, which can then be pyrolyzed into N-doped carbon in an Ar environment. The resulting Ag/ZnO@N-carbon has a larger particle size than Ag/ZnO.

### N_2_ Adsorption-Desorption Isotherms Analysis

**FIGURE 3:**
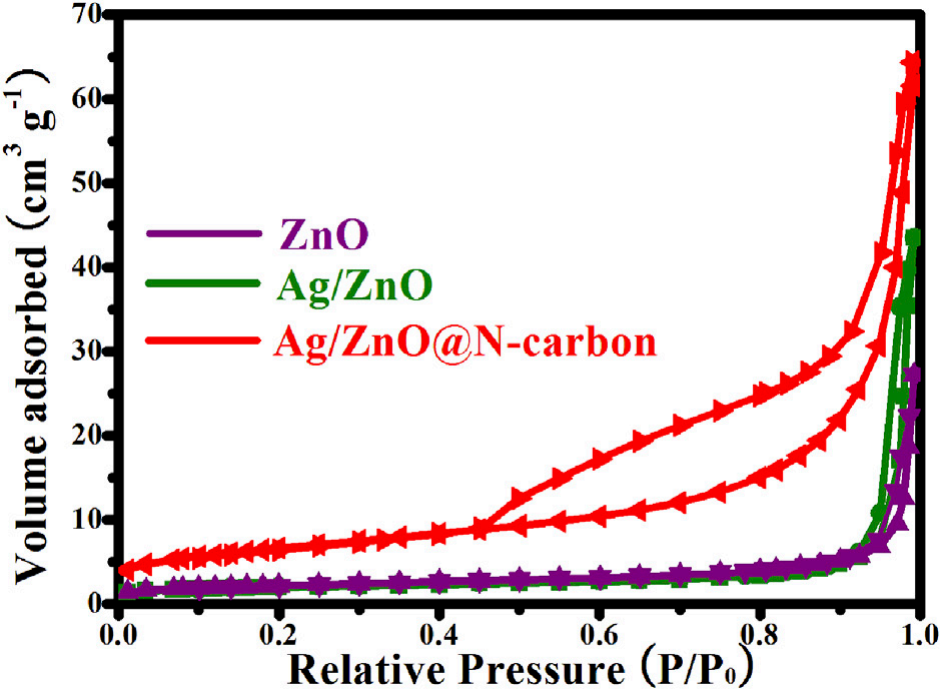
N_2_ adsorption-desorption isotherms of ZnO, Ag/ZnO and Ag/ZnO@N-carbon.

Figure 3 depicts the N_2_ adsorption-desorption isotherms for Ag/ZnO@N-carbon, Ag/ZnO, and ZnO. All of the samples’ N_2_ adsorption-desorption isotherms are assigned to a typical IV type isotherm with an H1 hysteresis loop, demonstrating adsorption at low pressure and multilayer adsorption at high pressure. It establishes the mesoporous structure of Ag/ZnO@N-carbon, Ag/ZnO, and ZnO. The BET method was applied to determine the specific surface areas of all samples. The specific surface area of Ag/ZnO@N-carbon is 22.91 m^2^/g, which is greater than that of ZnO (6.30 m^2^/g) and Ag/ZnO (7.24 m^2^/g). It is believed to be caused by porous N-doped carbon.

### X-Ray Photoelectron Spectroscopy Analysis

**FIGURE 4:**
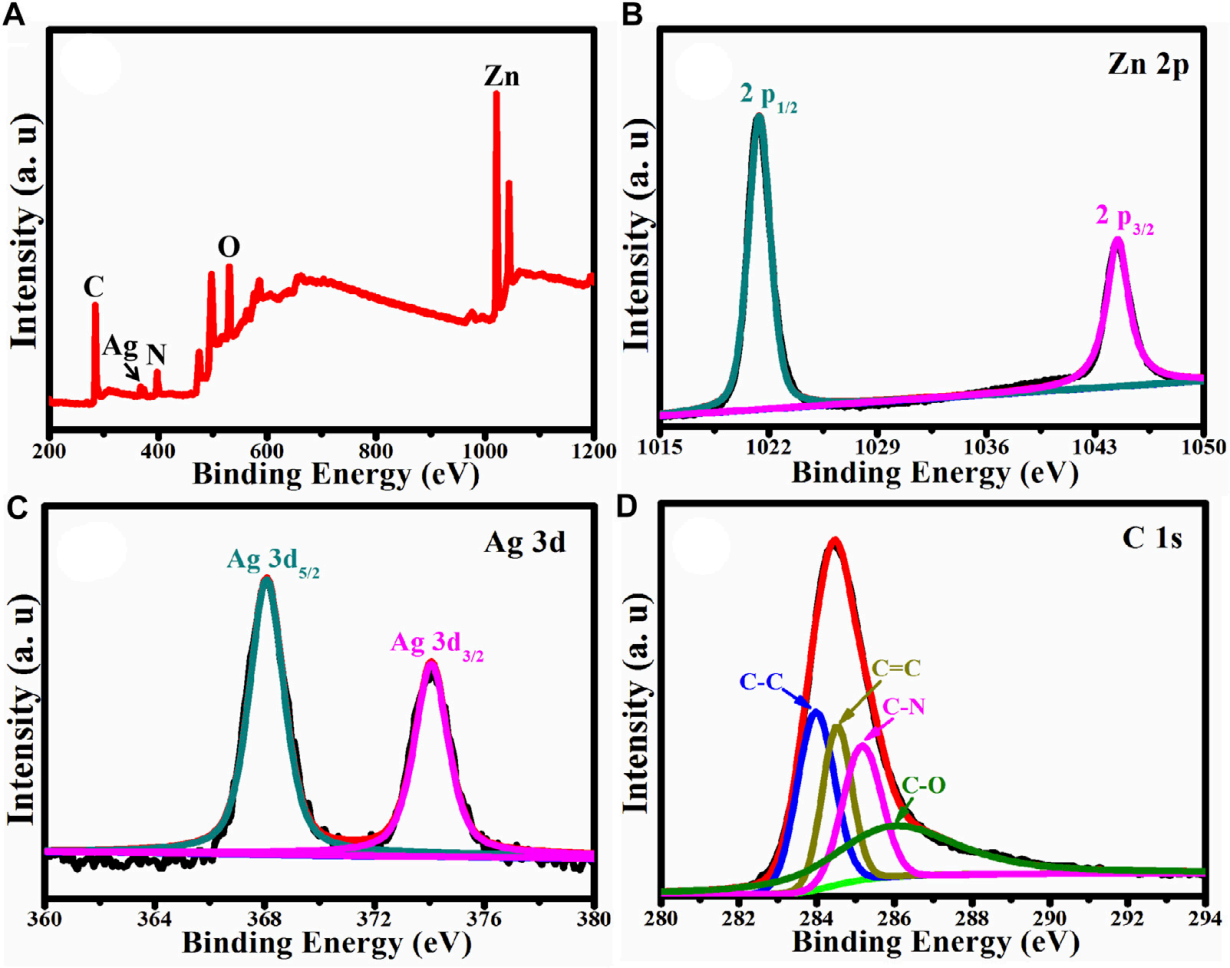
**(A)** XPS survey spectrum of Ag/ZnO@C and the corresponding high-resolution spectra of **(B)** Zn 2p, **(C)** Ag 3d, **(D)** C 1s.

X-ray Photoelectron Spectroscopy was used to analyze the chemical composition and chemical state of Ag/ZnO@N-carbon (XPS). Figure 4A shows the XPS survey spectrum of Ag/ZnO@N-carbon, which contains recognizable Ag, Zn, O, N, and C peaks. Zn 2p, Ag 3d and C 1s high-resolution spectra in Ag/ZnO@N-carbon are exhibited in Figures 4B–D, respectively. The high-resolution Zn 2p spectrum (Figure 4B) reveals two distinct binding energy peaks at 1,021.38 and 1,044.48 eV, which correspond to Zn 2p_3/2_ and Zn 2p_1/2_, respectively, demonstrating the existence of ZnO in the hexagonal wurtzite structure (Jin and Liu, 2020). Figure 4C is the high-resolution Ag 3d spectrum, which shows two binding energy peaks at 368.08 and 374.18 eV, indicating the successful doping of Ag in Ag/ZnO@N-carbon. C 1s has four distinct peaks at 283.98, 284.48, 285.18, and 286.08 eV, corresponding to the functional groups C-C, C=C, C-N, and C-O, respectively (Atchudan et al., 2018).

### Electron Transmission Microscopy Image Analysis

**FIGURE 5:**
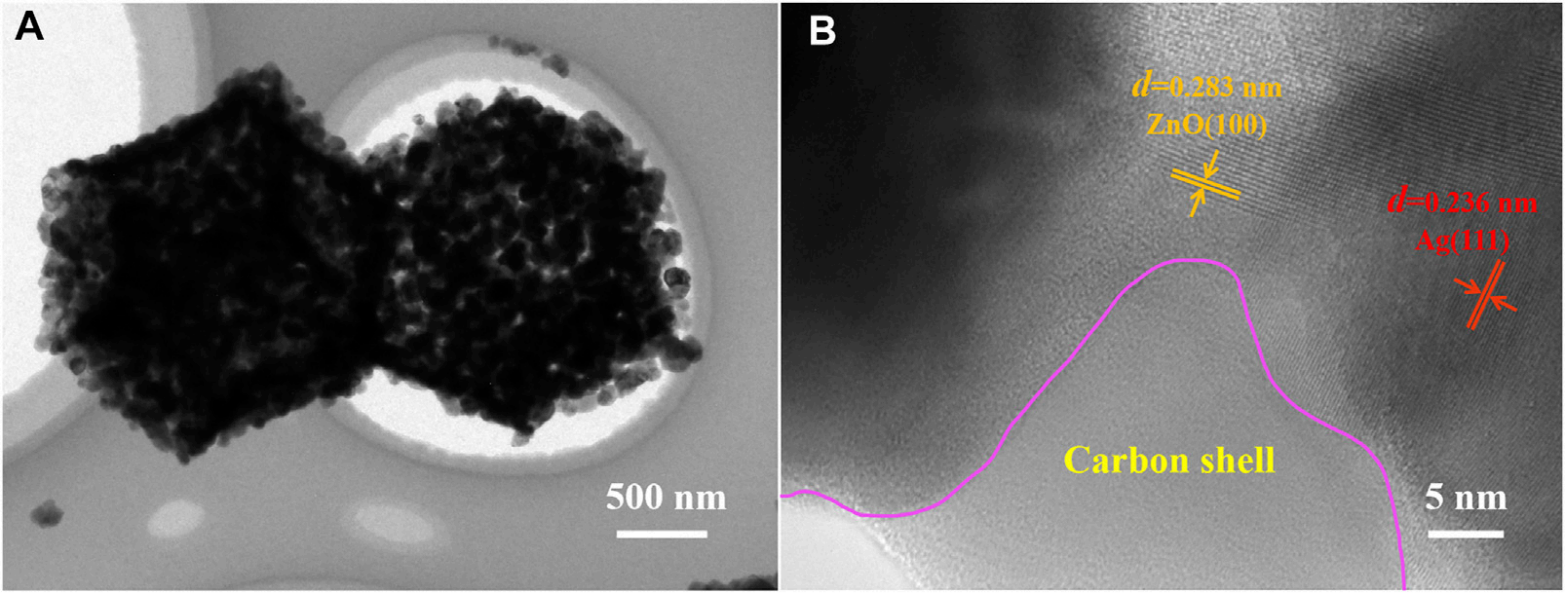
**(A)** Low-magnification TEM image and **(B)** high-magnification TEM image of Ag/ZnO@N-carbon.

Figure 5 shows a low-magnification TEM picture of Ag/ZnO@N-carbon and a high-magnification TEM image of Ag/ZnO@N-carbon. Following its SEM image, Ag/ZnO@N-carbon exhibits the polyhedral structure, as illustrated in Figure 2D. The TEM picture of Ag/ZnO@N-carbon in Figure 5B shows two distinct fringes with a lattice spacing of 0.236 and 0.283 nm, corresponding to the Ag (111) and ZnO (100) planes, respectively (Su et al., 2008; Thakur and Mandal, 2021). It confirms that ZnO is successfully doped with Ag. Furthermore, Ag-doped ZnO is coated with a layer of a porous structure. According to the above analysis, this porous structure is N-doped carbon.

### TG Curve Analysis

**FIGURE 6:**
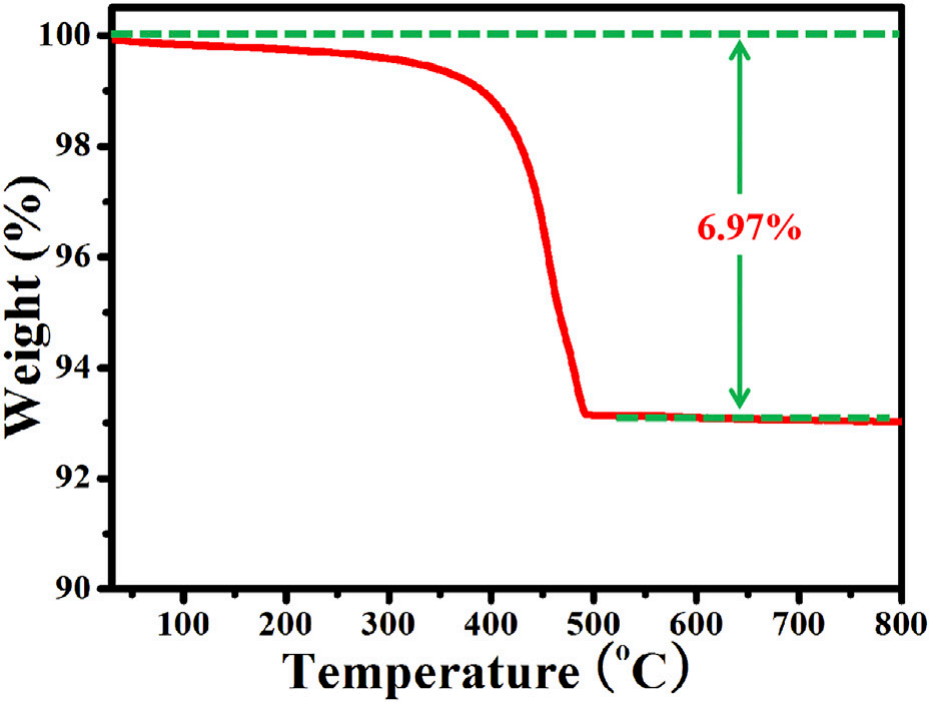
TG curve of Ag/ZnO@N-carbon under air atmosphere.

TG curve of Ag/ZnO@N-carbon was obtained under air atmosphere from 30 to 800°C with the heating rate of 5°C/min. As shown in Figure 6. Ag/ZnO@N-carbon exhibits a slight weight loss at relative low temperature, which belongs to the adsorbed water in Ag/ZnO@N-carbon. When the temperature is heated to 250°C, Ag/ZnO@N-carbon shows obvious weight loss. According to the BET, XPS and TEM analysis, it confirms that Ag/ZnO@N-carbon contains N doped porous carbon. The obvious weight loss of Ag/ZnO@N-carbon from 250 to 460°C is attributed to the oxidize N-doped porous carbon. It can be clearly seen that the weight loss of Ag/ZnO@N-carbon is 6.97%. The weight loss of pure N-doped porous carbon in air atmosphere is 65.34%. It can be calculated that the content of N-doped porous carbon in Ag/ZnO@N-carbon is about 10.67%.

### UV-Vis Absorbance Spectra Analysis

**FIGURE 7:**
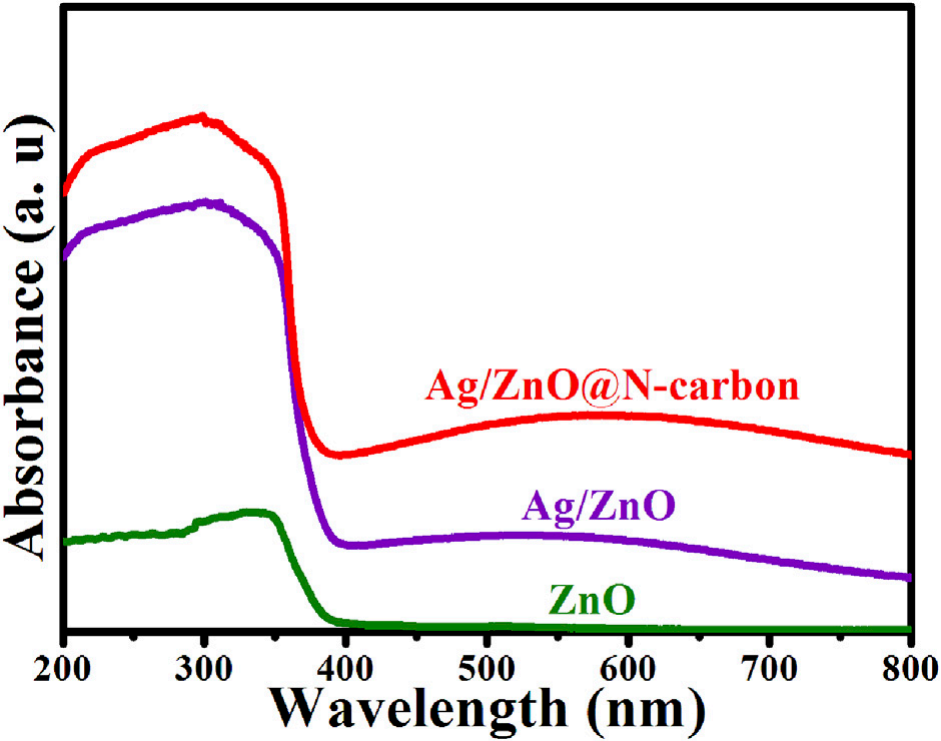
UV-vis absorbance spectra of ZnO, Ag/ZnO, Ag/ZnO@N-carbon.

The UV-vis absorbance spectra were used to analyze the photo-response of ZnO, Ag/ZnO, Ag/ZnO@N-carbon. The bandgap energy of semiconductors can be calculated by E_g_ = 1,240/λ formulae (where: “E_g_” represents the bandgap energy and “λ” the absorption edge, respectively) (Liu et al., 2018). As illustrated in Figure 7, all samples exhibit a decisive absorbance step in the wavelength range of 350–400 nm, corresponding to ZnO’s distinctive peak. The bandgap energy is 3.2 eV. Ag/ZnO and Ag/ZnO@N-carbon, on the other hand, exhibit an extensive absorbance range between 400 and 800 nm. It is due to Ag doping, which can introduce free carriers and alter the Fermi level in the conduction band, resulting in a substantial increase in photocatalytic performance.

### Photocatalytic Degradation Properties

**FIGURE 8:**
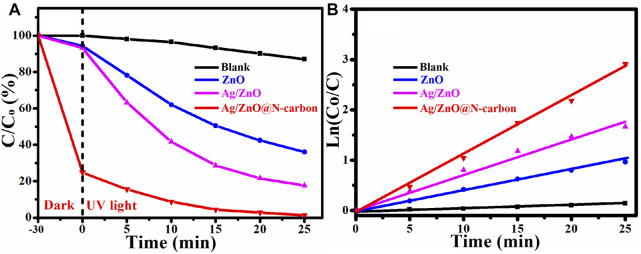
**(A)** Photocatalytic degradation and **(B)** the corresponding kinetics of all synthesized sample under UV light.

Degradation of RhB under UV irradiation at room temperature was used to assess the photocatalytic activity of ZnO, Ag/ZnO, and Ag/ZnO@N-carbon. The full procedures were carried out in the same manner as the catalytic activity tests. According to Figure 8A, after 30 min of dark reaction, Ag/ZnO@N-carbon can adsorb 75.17% of RhB, which is higher than ZnO (5.64%) and Ag/ZnO@N-carbon (6.82%). It could be attributable to Ag/ZnO@N-large carbon’s specific surface area. The entire reaction vessel was then subjected to UV light. Ag/ZnO@N-carbon can degrade 98.65% after 25 min of UV light irradiation, which is higher than ZnO (63.42%) and Ag/ZnO (82.36%). Under UV illumination, all produced samples show the same kinetics in Figure 8B kt is determined using the kinetic constant equation Ln (C_o_/C) = kt (k and t are the pseudo-first-rate kinetic constant and irradiation time). ZnO, Ag/ZnO, and Ag/ZnO@N-carbon have a kinetic constant of 0.0424, 0.0713, and 0.1112 min^−1^, respectively. The photocatalytic activity of Ag/ZnO@N-carbon is the highest. The outstanding photocatalytic activity of Ag/ZnO@N-carbon may be attributed to the excellent photo-degradation property of Ag/ZnO and the high adsorption performance of N-doped porous carbon.

**FIGURE 9:**
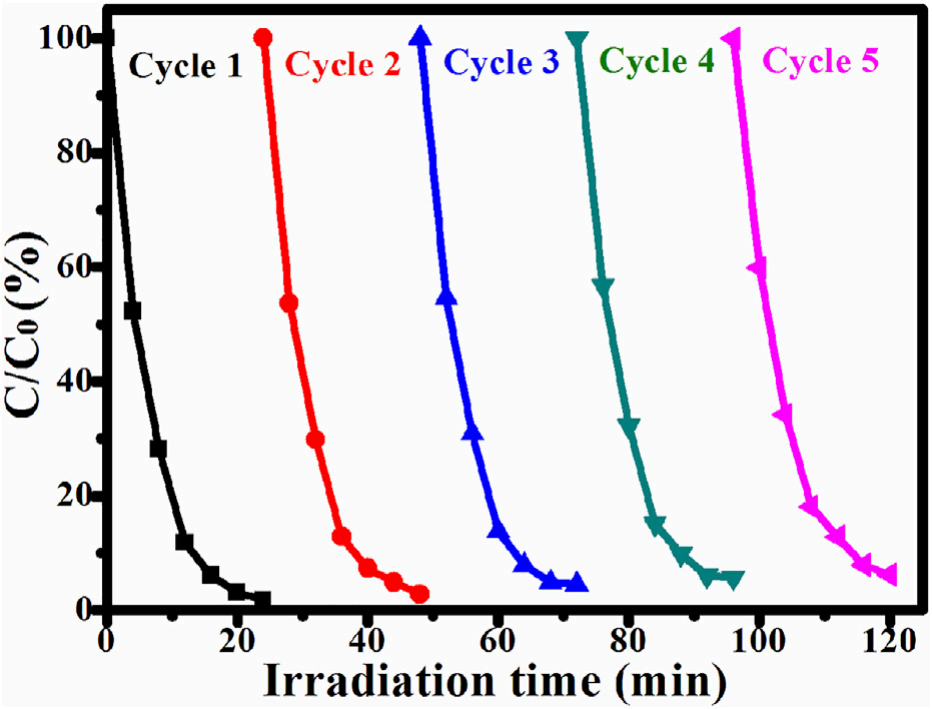
Efficiency of UV light driven degradation of RhB by Ag/ZnO@N-carbon for five runs.

Except for photocatalytic activity, stability is also another important property. Under UV light irradiation, Ag/ZnO@N-carbon was used to decompose RhB in this recycling experiment. Figure 9 depicts the result. For the fifth time in a row, the Ag/ZnO@N-carbon photocatalyst still performs admirably. There was a significant drop in the degradation rate of Ag/ZnO@N-carbon from 96.65% to 93.76%. The slight decrease may be attributed to the unavoidable loss of photocatalyst during the cycle processes.

**FIGURE 10:**
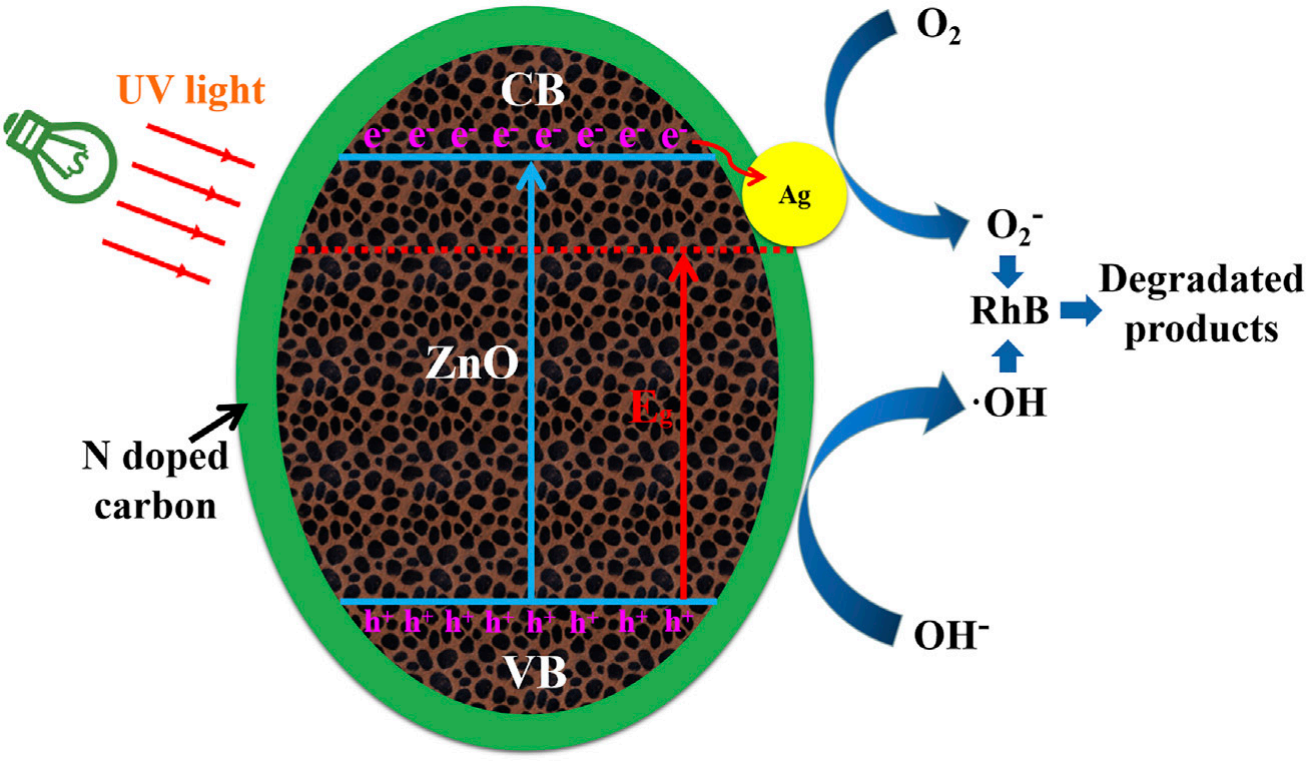
The possible schematic diagram of Ag/ZnO@N-carbon for degradation of RhB under UV light.

Under UV light irradiation, the photocatalytic activity and stability of Ag/ZnO@N-carbon photocatalyst for RhB degradation are superior to those of ZnO and Ag/ZnO. The breakdown of RhB by Ag/ZnO@N-carbon is schematically represented in Figure 10 under UV light. The bandgap energy of ZnO is 3.2 eV, which is less than the predicted value [ZnO’s valence band (VB) and conduction band (CB) are 2.86 and −0.34 eV, respectively] (Ding et al., 2019). When ZnO is doped with Ag, it exhibits photo-response for visible light. It may be attributed to the lattice distortion due to the doping of Ag ions in ZnO.

In addition, ZnO has a higher Fermi Level than Ag. The photo-generated electrons of ZnO can transfer to Ag easily. Doping Ag helps to separate electrons and holes from each other. In the photosynthesis process, electrons are captured by dissolved oxygen (O_2_), and the holes can mix with OH^−^ to produce OH. RhB decomposes into CO_2_ and H_2_O as a result of the formation of O_2-_ and OH. N-doped porous carbon absorbs RhB molecules during the photocatalytic degradation process, forming RhB molecules high concentration layer on its surface and pores. RhB molecules can also be degraded by O_2_^−^ and OH.

## Conclusion

In conclusion, Ag/ZnO@N-carbon was successfully synthesized by adopting PVP modified ZIF-8 as a precursor *via* adsorption, hydrothermal treatment, *in situ* growth, and carbonization processes and employed it as photocatalyst for degradation of RhB. When compared to ZnO, Ag doping can significantly improve ZnO’s photocatalytic activity. When Ag/ZnO is coated with an N-doped porous carbon layer, its adsorption ability for RhB increases dramatically from 6.82% to 75.17%. Under UV light irradiation for 25 min, Ag/ZnO@N-carbon has the maximum photocatalytic activity and can degrade 98.65% of RhB. More interestingly, Ag/ZnO@N-carbon shows high stability for RhB degradation. Ag/ZnO@N-carbon can similarly decompose 93.76% of RhB after five recycling trials. The outstanding photocatalytic performance of Ag/ZnO@N-carbon can be associated with the beneficial influence of Ag-doped ZnO’s excellent photocatalytic activity and N-doped porous carbon’s efficient adsorption.

## Data Availability

The original contributions presented in the study are included in the article/supplementary material, further inquiries can be directed to the corresponding author.
